# Energy-Efficient Hybrid Routing Protocol for IoT Communication Systems in 5G and Beyond

**DOI:** 10.3390/s21020537

**Published:** 2021-01-13

**Authors:** Mohammad Baniata, Haftu Tasew Reda, Naveen Chilamkurti, Alsharif Abuadbba

**Affiliations:** 1 Department of Computer Science and Engineering, Soongsil University, Seoul 06978, Korea; 2 Department of Computer Science and IT, La Trobe University, Plenty Rd., Bundoora, VIC 3086, Australia; h.reda@latrobe.edu.au (H.T.R.); n.chilamkurti@latrobe.edu.au (N.C.); 3 CSIRO’s Data61 and Cybersecurity Research, CSIRO, Marsfield, NSW 2122, Australia; sharif.abuadbba@data61.csiro.au

**Keywords:** wireless sensor network, Internet of Things, energy efficiency, routing protocol, 5G communication

## Abstract

One of the major concerns in wireless sensor networks (WSNs) is most of the sensor nodes are powered through limited lifetime of energy-constrained batteries, which majorly affects the performance, quality, and lifetime of the network. Therefore, diverse clustering methods are proposed to improve energy efficiency of the WSNs. In the meantime, fifth-generation (5G) communications require that several Internet of Things (IoT) applications need to adopt the use of multiple-input multiple-output (MIMO) antenna systems to provide an improved capacity over multi-path channel environment. In this paper, we study a clustering technique for MIMO-based IoT communication systems to achieve energy efficiency. In particular, a novel MIMO-based energy-efficient unequal hybrid clustering (MIMO-HC) protocol is proposed for applications on the IoT in the 5G environment and beyond. Experimental analysis is conducted to assess the effectiveness of the suggested MIMO-HC protocol and compared with existing state-of-the-art research. The proposed MIMO-HC scheme achieves less energy consumption and better network lifetime compared to existing techniques. Specifically, the proposed MIMO-HC improves the network lifetime by approximately 3× as long as the first node and the final node dies as compared with the existing protocol. Moreover, the energy that cluster heads consume on the proposed MIMO-HC is 40% less than that expended in the existing protocol.

## 1. Introduction

Recent advances in the field of wireless communications, electronics, and embedding system technologies have enabled the proliferation of large-scale wireless sensor networks (WSNs), with low-power, low-cost, multi-functional miniaturized wireless sensor nodes to communicate with each other using a radio frequency (RF) [1]. Such small sensor nodes, usually interconnected via an RF network and having a cooperative feature, are capable of sensing and controlling the intended environment, processing the collected data, and transmitting it back to a central station (CS). In particular, the convergence of WSNs and Internet of Things (IoTs) enables a ubiquitous interaction of things that have enormous applications both in private and public domains such as healthcare, industrial control systems, smart agriculture, smart cities, and environmental monitoring.

Fifth-generation (5G) mobile technology has become the standard that can handle the proliferation of IoT devices while maintaining the required quality of services. Furthermore, potential enablers of 5G such as Multiple-Input Multiple-Output (MIMO) [2] have the capability to improve performance, reduce energy consumption in transmission, and become more desirable for IoT devices. However, the wireless sensor nodes have resource limitations [1,3] such as memory, computation, and energy-constrained batteries. This has been one of the major problems in WSNs and related applications. Therefore, energy efficiency has been the prime goal when designing and deploying WSNs [4,5].

Wireless sensor nodes can be organized hierarchically into groups of clusters, to minimize energy consumption and provide a uniform distribution of load over the network [3]. Each cluster consists of Cluster Head that gathers data from the same cluster’s sensor nodes and transfers this data to a central station. Several clustering techniques are introduced in the WSNs, including the Low-Energy Adaptive Clustering Hierarchy (LEACH) [6], Power Efficient Gathering in Sensor Information Systems (PEGASIS) [7], and Energy-Efficient Unequal Clustering (EEUC) [8]. However, the unsolved issues of accelerated energy depletion, hotspot problem [9,10] in the previous clustering protocols for WSN should be addressed.

### 1.1. Motivation

The main motivation of this paper is the challenge of energy efficiency in 5G IoT systems as a result of managing data transmission of a staggering number of wireless sensor nodes. As discussed earlier in this section, various clustering protocols have been reported in the literature; however, most of these techniques are not viable for MIMO-based IoT communication system. Therefore, clustering protocols for conserving the energy of IoT systems in the 5G environment is still missing.

### 1.2. Contribution

To overcome the aforementioned challenge, a MIMO-based energy-efficient unequal hybrid clustering (MIMO-HC) routing protocol for IoT communication systems is proposed. In this method, data from the sensing area can be collected methodically, in which each sensor node must send the collected data to the central station in each round. Specifically, this article has the following major contributions.

1. We propose three algorithms for Cluster Head selection, hybrid cluster formation, and inter-cluster multi-hop routing. In particular, in the centralized-based MIMO-HC protocol, the central station determines each cluster’s head to reduce message transmission overhead. Furthermore, the proposed MIMO-HC divides the network hierarchically into unequal hybrid clusters to share the energy consumption among the cluster heads.
2. Clusters closer to the central station have small radius and their member nodes communicate with each other using chain topology to minimize the issue of overcrowding and collisions at cluster head. However, clusters that are farther away from the central station have a larger radius and their member nodes communicate with their respective cluster head using a single-hop topology to reduce delay when delivering data from source to destination.
3. A probability sub-optimal multi-hop routing mechanism among cluster heads is proposed to reduce the load on the cluster heads and to increase the lifespan of the sensor network. Finally, a ranking method is applied for choosing the proper communication interface.
4. We evaluate with existing techniques (namely UN-LEACH [11]), and the proposed protocol has a much better network lifespan. For example, in the proposed protocol, the first node and the final node die about 3 times as long as compared with the existing protocol. Moreover, samples of energy consumption by cluster heads have a standard deviation of $1.07\times 10^{-2}$ in the proposed protocol and those in the UN-LEACH have $3.16\times 10^{-2}$. In other words, the proposed protocol has a better load balancing capability than the UN-LEACH protocol.

### 1.3. Paper Outline

The remainder of the article is organized as follows. In Section 2, related works is presented. The next section discusses problem formulation and existing challenges. In Section 4, proposed methodology and employed algorithms are presented. Furthermore, Section 5 discusses performance evaluations and simulation results of the proposed methodology. Finally, the paper is concluded in Section 6.

## 2. Related Works

Several clustering protocols [6,7,12,13,14] and unequal clustering protocols [8,11,15,16,17,18,19,20] have been proposed to lessen the energy depletion.

The authors in [6] proposed LEACH, a protocol that uses control mechanism and local coordination to establish cluster activities. LEACH is a distributed cluster formation in which nodes randomly select themselves as cluster heads without the control of central station or providing frameworks for data aggregation. Every sensor node picks an arbitrary value between 0 and 1 to become a Cluster Head. LEACH follows a randomized Cluster Head rotation to share the burden of energy between the sensor nodes. Another enhanced version of the LEACH scheme, LEACH-Centralized (LEACH-C), is suggested by [12]. As compared to LEACH, in LEACH-C a central station performs cluster formation and decides the cluster heads by using a simulated annealing technique. However, LEACH and LEACH-C have drawbacks including variable set of cluster formation, randomized and unequal cluster heads deployment resulting in a short-lived network. This is due to the inefficient consumption of energy. Various types of LEACH protocols have been proposed to address these issues. Lindsey and Raghavendra [7] propose PEGASIS as a means to reduce issues of overhead in WSNs. PEGASIS uses greedy algorithm to form chains by neighboring nodes. The Cluster Head of the chain is assigned to forward data to the central station instead of several nodes. Sensors in the chain rotate among each other to forward data to central station. Yet, one of the disadvantages of PEGASIS is that whole data transmission to the central station takes longer due to the greedy chain forming process, which results in a higher latency. An energy-efficient Cluster Head selection scheme EECHS [13] protocol is suggested to lessen the energy depletion caused by Cluster Head selection. In EECHS there are three types of nodes within a cluster: Cluster Head, Member node, and scheduling node. The scheduling node is intended to track and store real-time information about the remaining energy of all nodes within cluster. In the Cluster Head selection phase, the scheduling node specifies a corresponding Member node as the new cluster head based on the monitored results.

EEUC [8] has been developed to address the problems associated with WSN hotspots. EEUC organize the network into unequal radius cluster, where clusters adjacent to the central station have smaller radius than those far from the central station. Therefore, Cluster Head adjacent to the central station saves power for data routing in multi-hop route. EAUCF [16] is an unequal clustering mechanism which employs the fuzzy techniques, and considers the position of the central station when a cluster group has been created. According to the remaining energy level and range to the central station, EAUCF changes the radius of the Cluster Head. A particle swarm optimization-based unequal and fault tolerant clustering protocol (PSO-UFC) [19] protocol is suggested. PSO-UFC employs the unequal clustering method to balance intra and inter-cluster energy depletion among the master cluster head. In addition, in PSO-UFC protocol the network connectivity is recovered by selecting an additional Cluster Head called surrogate Cluster Head in case of unexpected failure of master Cluster Head.

A flooding tree uneven clustering FTUC [20] protocol is suggested. FTUC developed for large-scale networks. FTUC first determines minimum and maximum distances values of the network by constructing sub-network style tree and then apply the unequal cluster mechanism. Referenced position circles is defined to equally elect cluster heads. Cluster heads are therefore selected based on the remaining energy of the node and their distance to the referenced circle.

A gradient-based routing (EBCAG) protocol [15] is suggested to address constraints resulting from unequaled energy depletion among cluster heads. EBCAG divides the networks into unequal diameter cluster and manages the distribution of cluster heads via hop counting. Every sensor in EBCAG holds a gradient value specified as the lowest possible hop-count to central station. Ren et al. [11] suggested an approach with unequal sized clusters based on LEACH for significantly improved power usage and prolonged network lifetime. Selection of Cluster Head is determined by two factors, namely the energy ratio and the competition distance. Under this approach, depending on the obtained distance matrix from the central station, sensor nodes will change their sending power.

Energy-Aware Distributed Unequal Clustering (EADUC) [17] protocol is an uneven hierarchical clustering strategy accommodating both homogeneous and heterogeneous networks. The creation of clusters is categorized into three stages: the stage of obtaining neighboring node information, the Cluster Head selection stage, and cluster formation stage. Cluster heads with greater remaining energy and distance from the central station would have a wider cluster diameter. Yang and Zhang [18] in their research proposed Energy Balancing Unequal Clustering Protocol (EB-UCP), where sensor node much nearer to the central station are more likely to be chosen as Cluster Head. In EB-UCP protocol, network is divided into layers, and nodes within each layer have their own probability of being selected as a Cluster Head. Table 1 shows the comparison between our proposed method and existing state-of-the-art literature. Accordingly, compared to the existing ones the proposed method has several unique advantages including a much better load balancing capability, lower transmission overhead, and provides a fault tolerance feature.

## 3. Problem Formulation and Existing Challenges

Using IoT systems in a 5G environment, many difficulties and issues in the clustering approach need attention. This section addresses the challenges and the model of the network employed in our research.

### 3.1. Overview of WSN and IoT Communication Systems

WSNs have gained considerable attention in research and industrial use, due to their simple features and economic viability. WSN implementation usually requires countless embedded and networked wireless sensor nodes to gather data on the physical phenomenon and to disseminate to the intended application. The wireless sensor nodes, however, typically have design and resource limitations [1,3] such as memory, computation, and power although these limitations can vary somewhat among different applications. In particular, WSNs are powered with energy-constrained batteries and it is difficult to recharge their batteries once again after they are diffused in the sensing field.

#### 3.1.1. Basic Clustering in WSNs

A mechanism known as clustering is used to segment the entire topology of WSN into sub-regions for efficient data transmission and prolonging of network duration. Typically, every cluster consists of a Cluster Head, which collects data already sensed by the member nodes inside the cluster and returns the collected data to the central station. The communication between sensor nodes within a cluster is described as intra-cluster routing, while the communication between separate clusters is known as inter-cluster routing. In WSNs, to collect the data and to pass it on to the central station, single-or multi-hop communication is implemented as a routing paradigm. The Cluster Head responsibility is logically rotated among each member within a cluster to achieve longer network lifetime and to reduce the depletion of energy for the WSN.

#### 3.1.2. Accelerated and Unbalanced Energy Consumption Issue

Cluster Head consumes more energy over a large-scale network than its member nodes. This is because it collects and aggregates data from nodes within cluster and transfers the same data to the central station over a long distance. Therefore, clustering algorithms divide the process into rounds to equalize the energy among sensor nodes, and rotate the cluster head responsibility among the nodes inside a cluster. Nevertheless, the consumption of energy between cluster heads remains unbalanced due to multi-hop route among multiple cluster heads, in which a Cluster Head near the central station serves as a router for the rest of the sensors and the relay traffic will be overloaded. Thus, some nodes die prematurely, leading to network holes. The optimum route among the cluster heads is another issue in the multi-hop communication model, using this route for any data transmission would contribute to the energy drainage among the cluster heads.

#### 3.1.3. Clustering in IoT Communication System

The use of clustering approaches for IoT communications under the 5G environment is determined by analyzing the main differences between WSNs and IoT based on different features. First, while WSNs were originally established for monitoring and collecting data for an exclusive environment such as military surveillance [21], health monitoring [22], IoT communication systems are applied in heterogeneous environments including homes, buildings, smart cities [23], and smart grids [24,25,26,27]. Following their applications, the amount of data transmitted in the WSNs is relatively smaller than in the IoT communication systems, which require a very high bandwidth to accomplish their goal. Furthermore, while the wireless sensor nodes under the WSNs are generally assumed to be homogeneous in terms of their battery, memory, sensing, and processing capability, senor nodes in IoT applications are heterogeneous and are no longer restricted to sensor basic features, rather they have diverse characteristics and functionalities. Another attribute that makes WSNs different from IoT devices is the communication characteristics: communication range, communication band, and the consumption of transmission power.

### 3.2. Inherent Challenges on the Use of MIMO in 5G IoT Communication System

Currently, a range of wireless communication technologies such as ZigBee, Bluetooth, Wi-Fi, LTE, Bluetooth, LoRa, etc. are contending to be used in IoT applications [1]. Each communication technology has its own features, including data transfer range, data rate, depletion of energy, and operating frequency. A few communication technologies are likely to deplete less energy than others (which usually corresponds to a lower data rate). Recent studies have shown that by using MIMO in the transmitter and the receiver, numerous data streams which are independent can be multiplexed at certain frequency-time window [28]. Accordingly, the multiplexing of various signals over separate antennas increases the probability of data rate of a link. This is achieved through the propagation of many highly correlated signals over independent fading channels. Yet, data transmission is still a challenging factor in IoT systems. When routing, the nodes’ data should consider the following factors.

1. The distance among nodes and service requirements on the network.
2. The transmission restriction given for the radio access technology.

### 3.3. System Model and Assumptions

We assume a wireless network of *N* number of IoT smart sensors which can be distributed evenly over M×M square region to periodically observe the environment. Let $s_{i}$ is the $i^{th}$ sensor then the corresponding set of sensor nodes is given by *S* = {$s_{1}$, $s_{2}$, …, $s_{n}$}|${}_{n=1}^{|S|=N}$.

Our proposed system model is based on the following eight assumptions.

1. All wireless sensor nodes support MIMO.
2. Each wireless sensor node sends only small volume of data. However, the Cluster Head can send big volume of data.
3. All sensor nodes have unequal capabilities (communication, processing, and battery)
4. Energy is limited (no continuous power supply).
5. All sensor nodes are not provided with a GPS-capable device (i.e., they are assumed as location-unaware).
6. The nodes can control their transmission power level depending on the distance to the receiver.
7. Nodes are assumed to be stationary.
8. Each IoT device has various options on communication technologies for data transfer symbolized as $NAI_{i}$ (see Section 4.4).

A similar wireless transmission/reception model is adopted as described in [12]. In this case, the wireless system diffuses energy to render the transmitter and amplifier of the transmitter. In addition, the wireless system emits energy for operating of the receiving circuit. Also, the power loss ($d^{2}$) under the free space model and the power loss ($d^{4}$) model under the multi-path fading have been used, where the energy loss is dependent on the distance between the transmitting end and the receiving end to power the transmitter. The power consumed to convey an *l*-bit message for a distance *d* is calculated in this radio model as:

Transmitter side:

$E_{TX}$(l, d) = $E_{TX-elec}$(l) + $E_{TX-amp}$(l, d)

(1) $$=\left\{\begin{array}{c} {\mathrm{lE}}_{\mathrm{elec}}+E\epsilon_{\mathrm{fs}}d^{2},d<d_{0}\\ {\mathrm{lE}}_{\mathrm{elec}}+E\epsilon_{\mathrm{mp}}d^{4},d\geq d_{0} \end{array}\right.$$

Receiver side:

(2) $$E_{\mathrm{RX}}=E_{\mathrm{RX}-\mathrm{elec}}\left(l\right)={\mathrm{lE}}_{\mathrm{elec}}$$

## 4. Proposed Methodology

Throughout this section, we explain the proposed approach and the algorithms employed. Justifications for the methodology are also described.

**Figure 1:**
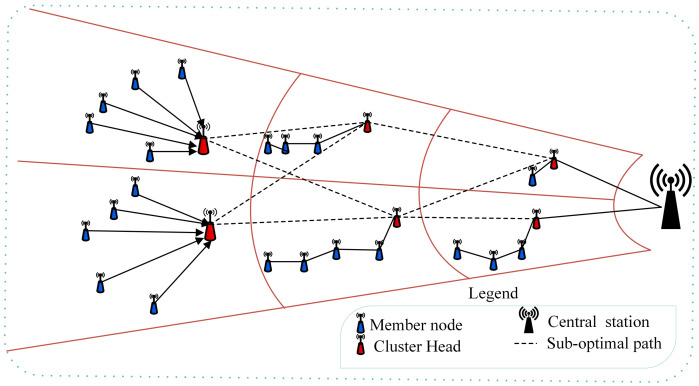
Overview of the proposed MIMO-HC technique.

The proposed MIMO-HC protocol comprises three stages: (1) Cluster Head selection, (2) Hybrid cluster creation, and (3) data gathering and propagation. These are discussed below. Figure 1 is the proposed architecture. From the figure the circles of variable diameter reflect the unequal clusters architecture with two types of communication topologies: single-hop and multi-hop (chain). In addition, routes that are linking the cluster heads represent the sub-optimal multi-hop routing model. As compared to the existing ones, the proposed method has several unique advantages including:

- a much better load balancing capability,
- lower transmission overhead, and
- supports a fault tolerance feature which is not present in the existing literature.

Justifications of the proposed methodology are provided in Section 5.

### 4.1. Cluster Head Selection Phase

**Figure 2:**
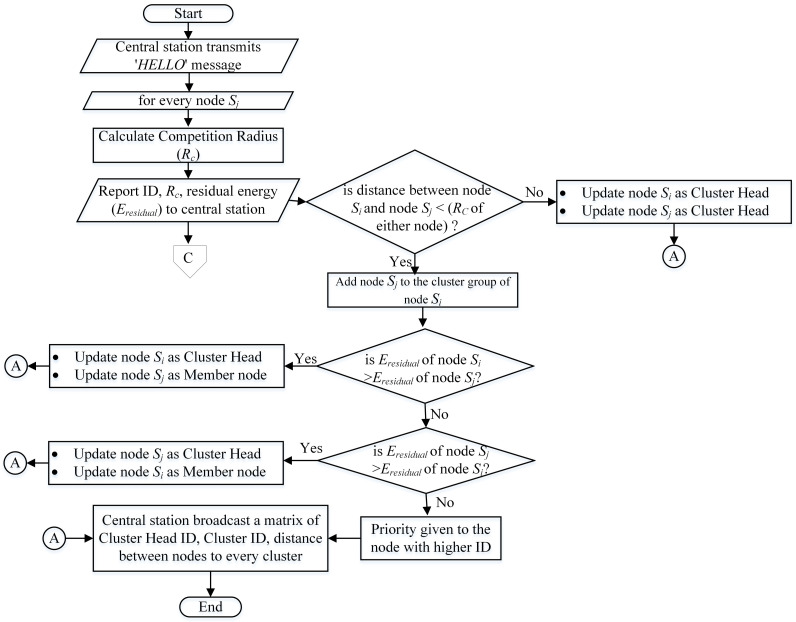
Selection of Cluster Head.

The algorithmic flow chart in Figure 2 demonstrates the selection process of Cluster Head and is explained as follows. With a certain power level, a “HELLO” message is transmitted by the central station to every sensor node positioned in the network area. Received signal strength helps every node to estimate its relative distance to the central station, and therefore, generates a hybrid of clusters of uneven size. Upon receiving such a message, nodes would easily compute their Competition Radius and deliver a report containing estimated current residual energy, altered Competition Radius, and the ID of node to central station. In our proposed MIMO-HC, the candidature to becoming the cluster head is determined only if a node contains higher leftover energy than nodes fall in its radius. To build a hybrid clusters with unequally size, every node should determine its own Competition Radius. In MIMO-HC, nodes that have greater remaining energy will handle additional tasks. Thus, the clusters, which are far from the central station with higher leftover energy, use single-hop topology and have greater number of nodes than those in the proximity which use chain topology. To do this, a sensor node with higher leftover energy needs to augment its own Competition Radius as its range to central station increases. On the one hand, nodes with lower residual energy should have a smaller Competition Radius to prevent their early death. The node Competition Radius is computed through the formula of Rc by (3).

(3) $$R_{C}=\left[1-\alpha \frac{(d_{max}-d\left(s_{i},CS\right)}{\left(d_{max}-d_{min}\right)}-\beta \left(1-\frac{E_{residual}}{E_{max}}\right)\right]R_{C}^{0}$$

where:

- *d* ($s_{i}$,CS)symbolizes the range from sensor node $s_{i}$ to central station,
- α and β are the weighted factors, where 0≤α≤1 and 0≤β≤1,
- $R_{C}^{0}$ symbolizes the maximal value of Competition Radius,
- $E_{residual}$ symbolizes the estimated current residual energy of node $s_{i}$.
- $E_{max}$ symbolizes the primary node energy, usually identical for all nodes.
- $d_{max}$ and $d_{min}$ represent the maximal and minimal range among both central station and sensor nodes.

Once the central station obtains the notification messages from the sensors, the ultimate Cluster Head is chosen among the candidate nodes. After that central station generates a matrix and sends it out to all sensors distributed on the network. The matrix

$$\left[\begin{array}{ccc} d_{11}^{h} & \ldots & d_{1n}^{h}\\ . & d_{ij}^{h} & .\\ d_{n1}^{h} & \ldots & d_{nn}^{h} \end{array}\right]$$

involves a list of nodes related to a particular cluster *h*, and the range among the nodes, and the Cluster Head ID where:

- $d_{ij}^{h}$ symbolizes the range among node *i* and node *j*.
- *h* symbolizes the Cluster Head ID.

Obtaining the matrix allows every sensor to know easily and accurately their respective Cluster Head and the range to other sensors; hence, the power of transmission can be changed according to the received distance to minimize the consumption of energy.

### 4.2. Hybrid Cluster Construction Phase

**Figure 3:**
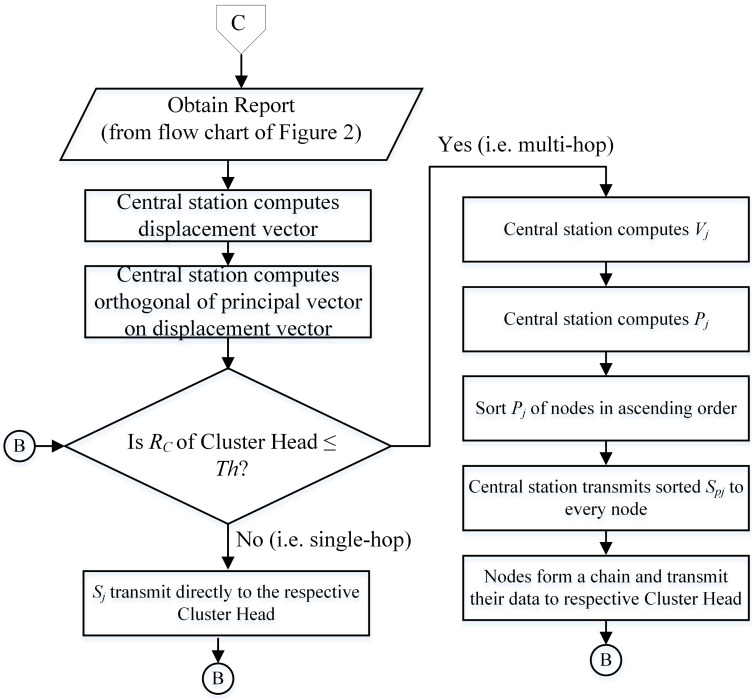
Formation of hybrid cluster.

Central station position impacts the cluster chain formation or structure of the network topology. Moreover, it affects the overall energy consumption of nodes. This will affect the quality and efficiency of the network. To alleviate the aforementioned problems, a procedure of chain construction (see Figure 3 for details) is proposed. This procedure allows the created chain topology to adapt to the diverse positions of the central station. In MIMO-HC a projection method is used to create the chain to avoid abnormal cluster chain topology.

Each node projects its displacement vector in every cluster and all nodes in each cluster are ordered by the principal value of the vector. The principal vector is defined as (4).

(4) $$\vec{v}=\left\{\begin{array}{c} \left(x_{M},0\right)\;\mathrm{if}\;x_{a}=0\\ \left(0,y_{M}\right)\;\mathrm{if}\;y_{a}=0\\ (-1,\frac{x_{M}}{y_{M}})\;\mathrm{otherwise} \end{array}\right.$$

where:

- *($x_{M}$, $y_{M}$)* refers to the position of midpoint (M) of network region.
- $x_{a}$ and $y_{a}$ are the x and y symbol for else vector known as $\vec{a}$ defined in (5), used to link the central station with the network midpoint (M) ,where ($x_{B}$, $y_{B}$) denotes the location of central station.

(5) $$\vec{a}=\left(x_{B}-x_{M},y_{B}-y_{M}\right)$$

Figure 3 demonstrates flow chart of the proposed cluster chain formation and the steps are summarized below.

1. All the report messages from the sensors distributed across a network are gathered by the central station.
2. The central station estimates the position of these distributed nodes according to the obtained signal strength.
3. Based on received Cluster Head Competition Radius value $R_{c}$, central station decides type of communication topology within certain cluster by identified threshold value *Th*.
4. if the Cluster Head competition diameter $R_{c}$> *Th*, nodes within cluster wait for random time if no matrix received from the central station, nodes communicate their data directly to their respective Cluster Head.
5. if the Cluster Head competition diameter $R_{c}$≤ *Th*, central station computes the chain construction as described in Section 4.2.
6. The central station computes $\vec{a}$ and $\vec{v}$⊥$\vec{a}$.
7. For each node in the cluster, central station computes the vector $\vec{s_{j}}$, which connects the sensor node $s_{j}$ with the midpoint of the network area.
8. The projection of $\vec{s_{j}}$ onto $\vec{v}$ is computed by the central station using the following Equation (6)

(6) $$P_{j}\left(s_{j}\right)=\frac{\vec{v}.\vec{s_{j}}}{|\vec{v}|}.$$

**Figure 4:**
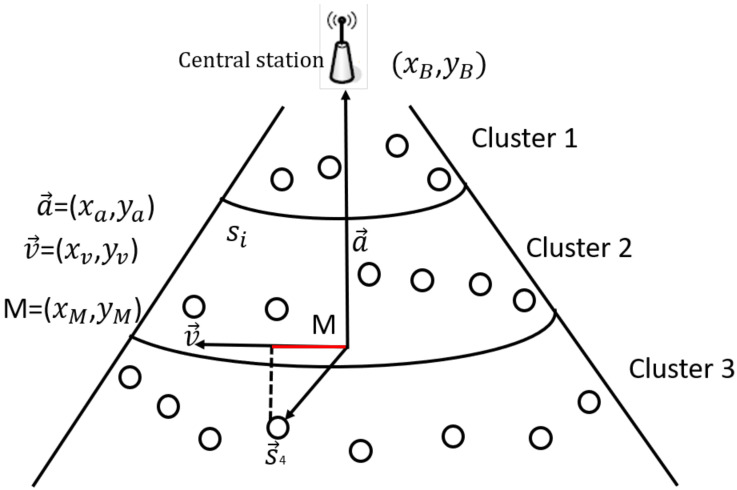
The method of projection for chain formation.

The central station constructs a matrix and transmits it to every sensor node when the node projection calculation concludes. The matrix includes ordered sensor nodes on every cluster according to the increasing value of the projection range. The obtained ordered projection value allows nodes on every cluster to build a chain schema. Figure 2 demonstrates the selection process of Cluster Head and is explained as follows Figure 4 is an illustration of the projection approach. Here, the vector for the sensor node $\vec{s_{4}}$ at the third cluster is calculated by central station. Moreover, it computes $P_{j}\left(s_{j}\right)$, which indicates the value of projection (marked by the red line color) of sensor node $\vec{s_{4}}$ onto $\vec{v}$ using (6). The same method applies to the other cluster nodes. Next, nodes in the cluster 3 are ordered according to the increasing value of the projection range and followed by construction of the chain topology.

### 4.3. Inter-Cluster Sub-Optimal Multi-Hop Routing

In this section, the proposed energy-aware, inter-cluster multi-hop routing method is discussed. This proposed method ensures that there are sub-optimal routes among cluster heads and the central station employed every once in a while for a connection.

**Figure 5:**
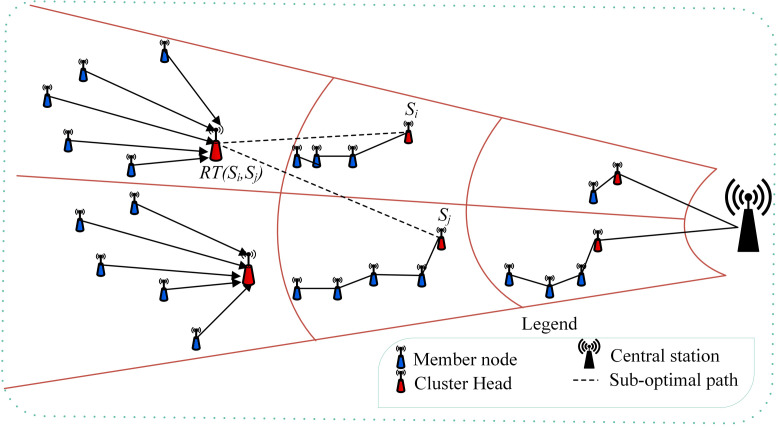
Description of inter-cluster sub-optimal multi-hop routing.

Selecting the optimum route among the cluster heads may lead to the consumption of energy levels across the route. This causes a considerable differences in the energy levels between nodes, which eventually induce partitioning of the network. Therefore, we have developed an energy-aware and a probabilistic multi-hop routing protocol to connect the cluster heads. Through the suggested MIMO-HC, we are attempting to detect several paths from the cluster heads to the central station, and the probability of selection is allocated to every route. Using the probability, one of the routes for each interval of data transfer from the Cluster Head to the central station is chosen randomly ($P_{s_{i},s_{j}}$), see Equation (10). Figure 5 describes the proposed inter-cluster sub-optimal multi-hop routing.

A simple energy metric, which was employed in [29], is adopted to meet our target. This metric provides details of the cost of using the cluster heads’ route and residual energy across the route (7).

(7) $$Cost_{ij}=e_{ij}^{c1}E_{residual_{i}}^{c2}$$

where

- $cost_{ij}$ symbolizes the cost metric among the cluster heads *i* and the Cluster Head *j*.
- $e_{ij}$ is the used energy for transferring and delivering on a route.
- $E_{residual}$ symbolizes the estimated current remaining energy of a Cluster Head.
- weighted factors represented by two symbols (*c*1 and *c*2).

**Figure 6:**
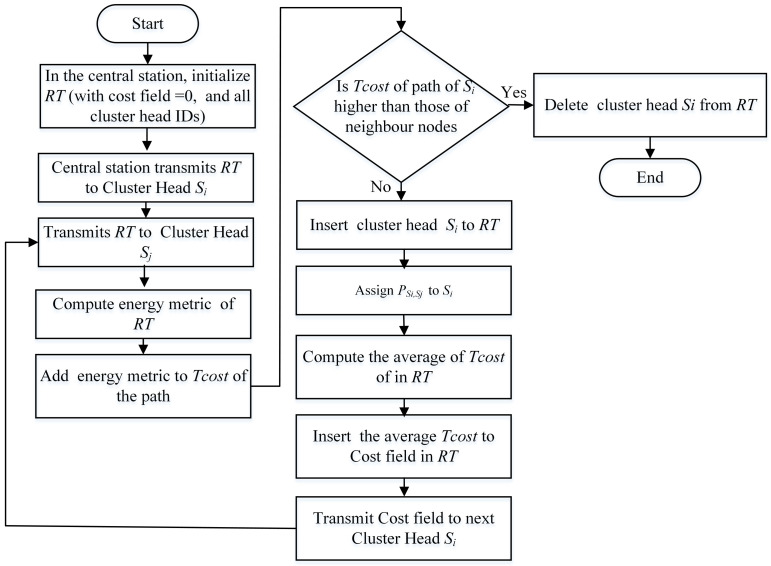
Inter-cluster sub-optimal multi-hop routing.

Figure 6 is the flow chart for the inter-cluster multi-hop routing topology. Upon identifying the Cluster Head and its belonging member nodes for the current running round, the central station creates a routing table (*RT*) and next broadcasts it to the intended Cluster Head. Before transmitting the generated routing table to the network, the central station attaches a set of Cluster Head IDs to the *RT* and at the same time determines the cost field in the *RT* with value zero (i.e., Cost(CS)=0).

Cluster Head $s_{j}$ calculates the energy metric for the transmitted *RT* by intermediate Cluster Head $s_{i}$ and adds it to the overall cost of the path (8)

(8) $$Tcost_{s_{i},s_{j}}=Cost_{s_{i}j}+e_{ij}^{c1}E_{residual_{i}}^{c2}.$$

Routes that have a very high cost are discarded and not added to the *RT*. Only the neighboring cluster heads $s_{i}$ with routes of low cost are added to the routing table $RT_{j}$ of Cluster Head $s_{j}$ as shown in Equation (9)

(9) $$RT_{j}=i|Tcost_{s_{i},s_{j}}\leq c1.(\underset{k}{min}\left(Tcost_{s_{k},s_{j}}\right).$$

A probability is given for every Cluster Head $s_{i}$ included in the $RT_{j}$ by the Cluster Head $s_{j}$, where this likelihood has an inverse relation to the cost.

(10) $$P_{s_{i},s_{j}}=\frac{\frac{1}{Tcost_{s_{i},s_{j}}}}{\sum_{k\in RT_{j}}\frac{1}{Tcost_{s_{k},s_{j}}}}$$

Later, the average cost of reaching central station via the adjacent cluster heads included in *RT* is computed by Cluster Head $s_{j}$.

(11) $$Tcost{(}_{s_{j}})=\sum_{i\in RT_{j}}Tcost_{s_{i},s_{j}}P_{s_{i},s_{j}}$$

Eventually, the current Cluster Head average cost is applied to the cost field and foreword it towards the next Cluster Head, this process continues to exist till the last Cluster Head is identified.

The proposed inter-cluster communication protocol specifies a Tx_Th threshold value such that when Cluster Head has a range less than or equivalent to Tx_Th to the central station, it conveys its data immediately to the central station.

### 4.4. Interface Selection

After the sub-optimal routes have been discovered between both the cluster heads and the central station and all nodes are available for communication, the sensor node should then be capable of determining suitable communication technology (interface) relying on its transfer requirements and communication capacities. In our proposed protocol, all sensor nodes, excluding the Cluster Head forward a small or large volume of data. All communication interfaces are permitted if the sensor node transfers a small volume of data. However, the communication interface with low energy loss must have the highest preference. High network bandwidth is necessitated when the sensor transfers a large volume of data. In such cases, mostly a communication interface with a data transfer rate greater than that provided by the system operating on sensor would be assigned for the ith Network Access Interface (*NAI*). The ranking Equation (12) [30] is used to reach this goal.

(12) $$Ranking_{i}=\frac{NAI_{i}^{r}}{{\left(NAI_{i}^{e}\right)}^{2}}$$

where:

- $NAI_{i}^{r}$ symbolizes the data transfer rate of the ith *NAI*,
- $NAI_{i}^{e}$ symbolizes the energy depletion for $NAI_{i}$ over a single unit communication range.

The interface communication range must also be taken into account by the node, whether it can reach the target node or the Cluster Head.

### 4.5. Route Maintenance

In intra-cluster multi-hop topology, adjacent nodes can check out each other using short noticing messages. If a node does not respond, the adjacent node considers it to be dead. The adjacent node will exceed the dead node and transmit data to the next sensor node which has been listed on the received matrix from the central station. Considering the inter-cluster multi-hop topology, our proposed technique guarantees the delivery of data between the cluster head members even if one of the communication links fails (see Figure 5). This is due to the sub-optimal technique in the proposed technique.

## 5. Performance Evaluation and Discussion

In this section, justifications for the proposed methodology and performance evaluations through numerical simulations are presented.

### 5.1. Justification of the Proposed Protocol

The following two points demonstrate the reason UN-LEACH protocol is chosen over the other protocols.

1. From cluster head selection perspective: To control the number of generated clusters, energy and Competition Radius are the main factors considered in our proposed protocol. These two factors are also inherent in the conventional UN-LEACH protocol.
2. From energy balance perspective: Similar to the UN-LEACH protocol, our proposed protocol is based on dividing the network into a hierarchical unequal clusters to balance the energy consumption among the cluster heads and to avoid hotspot issue that can be caused by overloading cluster heads closer to the central station.

However, the following two points are peculiar to our proposed technique which are not present in the UN-LEACH.

1. From topology perspective: Although the UN-LEACH protocol uses a single-hop topology for both the inter-cluster communication and intra-cluster communication, our proposed MIMO-HC protocol employs a mixed topology (i.e., a single-hop topology for the intra-cluster communication and multi-hop topology for the inter-cluster communication).
2. From scalability perspective: Our proposed technique aims to achieve improved network lifetime and better load balancing. Equally important, scalability of the proposed network model is investigated to illustrate its robustness to changes in the wireless network topology. Often, in the real-world, the network size can grow large, for example, due to a reasonable network expansion. For example, the authors of [14] have suggested K-means-based network partition algorithm to overcome multi-controller deployment issues in Software Defined Networking. Similar approaches can be used for a scalable process around the growth of wireless sensor nodes. Therefore, the proposed energy-efficient routing protocol should perform as well as at times where the network grows larger. For this purpose, the proposed technique should have the capability to support the scalability of the wireless network topology.

Therefore, due to the factors mentioned above, we believe that it sounds reasonable to implement to the proposed protocol. Accordingly, the performance evaluations of our proposed methodology are benchmarked against the UN-LEACH protocol.

In particular, to justify and evaluate the theoretical concepts of our proposed method several experiments have been carried out through MATLAB software tool. First, Cluster Head distribution and delay are investigated. In addition, our simulation results are compared with existing clustering algorithm UN-LEACH [11] in terms of energy consumption by the cluster heads, network lifetime, and the number of nodes alive when using the clustering protocols under the 5G environment. For our simulation environment, it is presumed that multiple wireless communication interfaces are usable on sensor device, where the sensor node can select between them according to its needs. The simulation environment is based on the system model as described in Section 3.3. We have simulated an area in which sensor nodes in the sensing area are equally distributed and the central station positioned outside the WSNs. A simulation round is defined as a time interval in which each senor node transfers a packet size of 25 bytes to the central station. The simulation result illustrates that the energy model is the same as the energy model used in [12]. The simulation parameters of the proposed MIMO-HC are shown in Table 2. We set α,β to 0.5, $R_{c}^{0}$ have a value between 0 and 200 in (3), Tx_Th = 130 m, and *c*1, *c*2 have value of 1, 50 respectively in (7). When the last node in the network dies, the simulation rounds stop counting. In addition, because of the irregular time death for the last node in the network, the number of rounds is varied. Therefore, the simulation results are recorded as an average of 30 runs for every simulation inputs/scenarios.

### 5.2. Cluster Head Distribution

**Figure 7:**
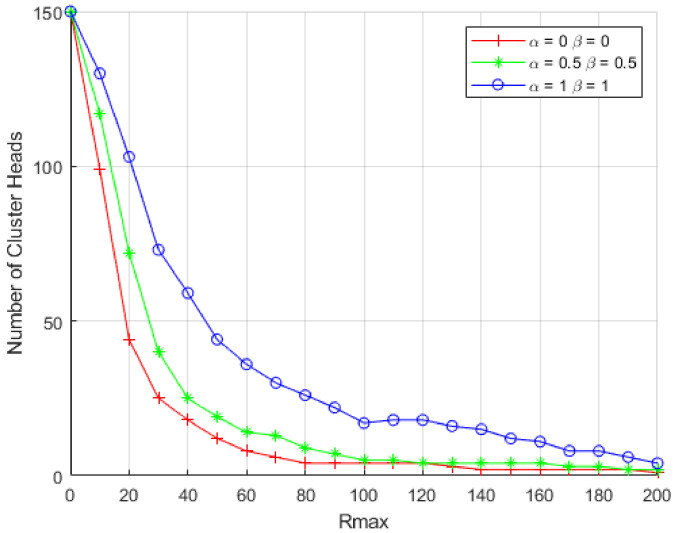
Number of Clusters Produced in the Network.

Here, we study the correlation among $R_{c}^{0}$ and the number of cluster heads when β and α take distinct values. Figure 7 shows the generated number of cluster heads in the network for the MIMO-HC. Figure 7 shows that if α and β are given a value of 0.5 each, the number of generated cluster heads would be smaller than α and β if their values are 1 each. Besides that, having α value of 0, and β value of 0 generates the least number of cluster heads. This is mainly attributable to the increase in the Competition Radius. In addition, with the decrease in radius, and α and β each with value of 1, the number of generated cluster heads increases. This implies the number of cluster heads produced is specified by $R_{c}^{0}$, α, and β.

**Figure 8:**
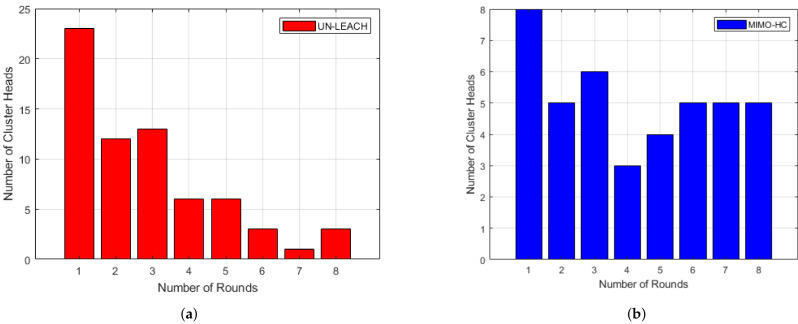
(**a**) Clusters distribution in UN-LEACH [11]; (**b**) Clusters distribution in MIMO-HC.

In our suggested MIMO-HC, we give α and β value of 0.5 each, and $R_{c}^{0}$ have value equal to 50. Figure 8 demonstrates the distribution of cluster numbers in both MIMO-HC and UN-LEACH. The numbers of clusters in MIMO-HC are steadier in contrast to UN-LEACH. This is attributed to the random technique that has been employed in UN-LEACH to create cluster heads, resulting in a totally changeable number of clusters.

### 5.3. Delay Analyses

**Figure 9:**
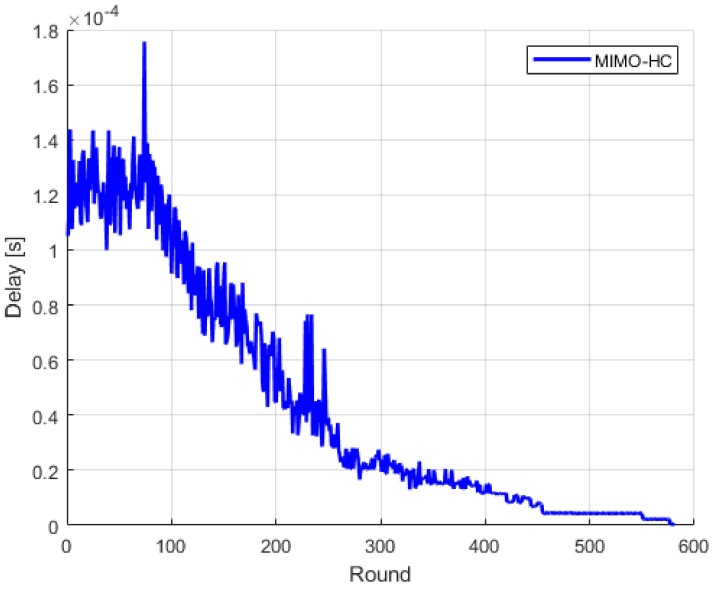
Delay.

Conventionally, nodes must route their data over all the attached nodes on the chain schema to reach their target. This further causes data propagation delay and redundant transmission. In contrast, by segmenting the whole network into uneven several clusters where clusters with small radius consist of short chains and clusters with large radius consist of single-hop topology, this attains less communication delay. In Figure 9, we can see that MIMO-HC has a good data delivery time.

### 5.4. Energy Efficiency

**Figure 10:**
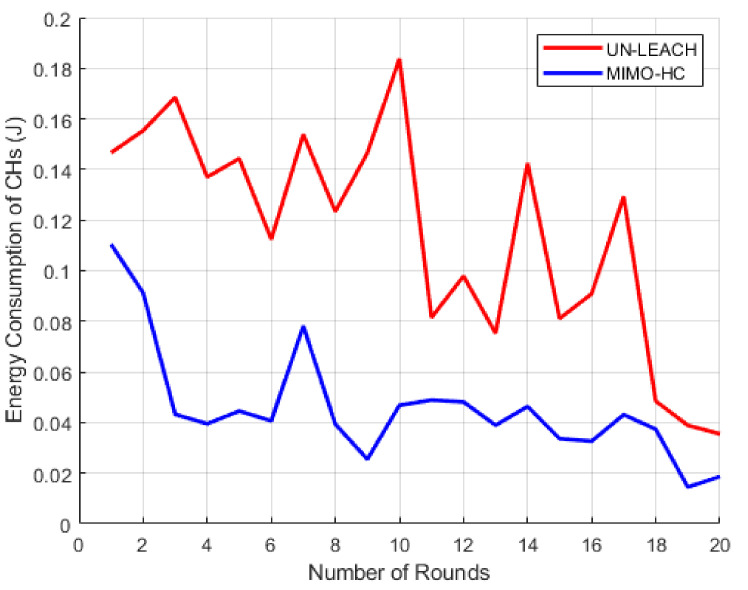
The amount of energy consumed by cluster heads.

**Figure 11:**
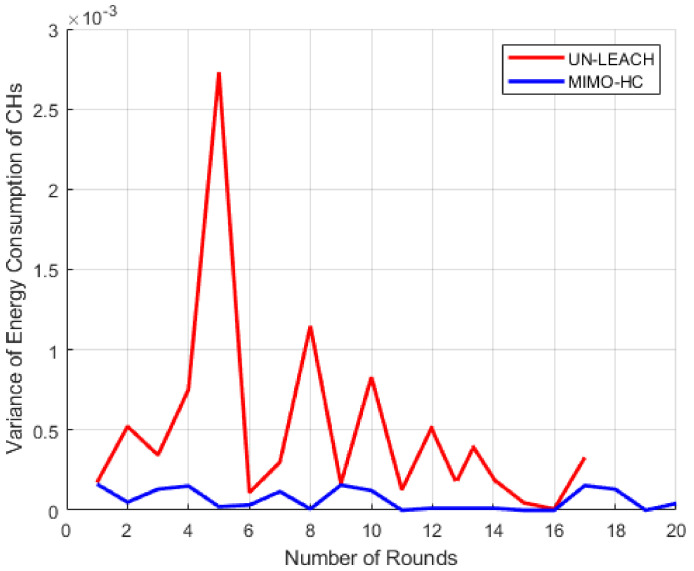
Divergence of energy depletion among cluster heads.

Here, the energy efficiency of the proposed MIMO-HC is analyzed. Figure 10 shows the energy cost that the cluster heads expense at MIMO-HC and UN-LEACH algorithms. The horizontal axis displays 20 sampled rounds of simulation, while the vertical axis displays the cost of energy expended by the cluster heads. The findings reveal that the energy that cluster heads consume on MIMO-HC in every round is much lower than when using UN-LEACH. Furthermore, by looking at Figure 11 the MIMO-HC equalized the depletion of energy among the cluster heads better than UN-LEACH. This happens due to cluster heads convey data in the UN-LEACH direct to the central station, and that incurs extra energy. Another factor is in the UN-LEACH method the cluster heads are not spread equally all over the network because of the random selection of the cluster heads.

**Figure 12:**
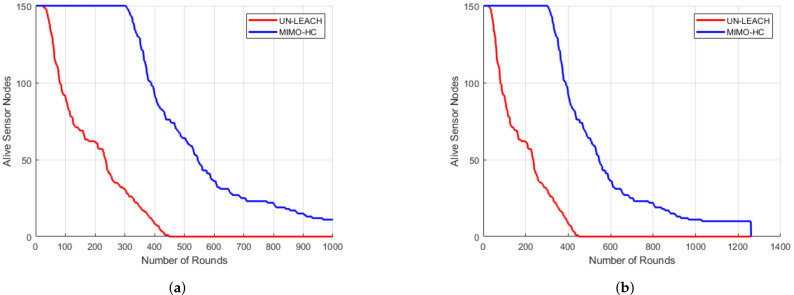
Network lifetime. (**a**) Time when first node perishes; (**b**) Time when last node perishes.

By contrast, cluster heads in MIMO-HC communicate with the central station through mixed hop (i.e., multi and single hops), and use sub-optimal routes among the cluster heads and the central station. As such, they transmit data via sub-optimal paths chosen at random which enable them to save a considerable amount of energy. Furthermore, the energy efficiency of our proposed model is investigated by evaluating the lifespan of the network (see Figure 12). Figure 12 displays the time at which the first and the final nodes die. As compared with the UN-LEACH, the MIMO-HC improves the time until both the first node and the final node die.

Figure 8 shows that the number of clusters in the MIMO-HC is steadier than in the UN-LEACH. Additionally, Figure 12 shows that the number of clusters in MIMO-HC has longer stability period than the UN-LEACH, where all the nodes in the MIMO-HC remain alive for 300 rounds and 50 rounds for the UN-LEAH. Consequently, the MIMO-HC has stable type of cluster in each round. This is due to the MIMO-HC can generate unequal clusters (using Equation (3)). In addition, using a *Th* value can ensure that clusters closer to the central station with small radius will use multi-hop topology and clusters far away from the central station with large radius will use single-hop topology.

Considering a sensing area of 200 m × 200 m, the UN-LEACH graph shows that sensor nodes die earlier as compared to MIMO-HC due to thinly scattered sensor nodes, which consume more energy for data transfer over large distance to their own Cluster Head. In addition, Cluster Head in UN-LEACH deplete additional energy to relay the data immediately to central station over a large distance. Contrary to the existing technique, the proposed MIMO-HC overcomes these drawbacks by carrying data to the particular Cluster Head using mixed chain and single-hop schema. Moreover, nodes at a distant location from the central station with little remaining energy are not determined to be the final Cluster Head.

**Figure 13:**
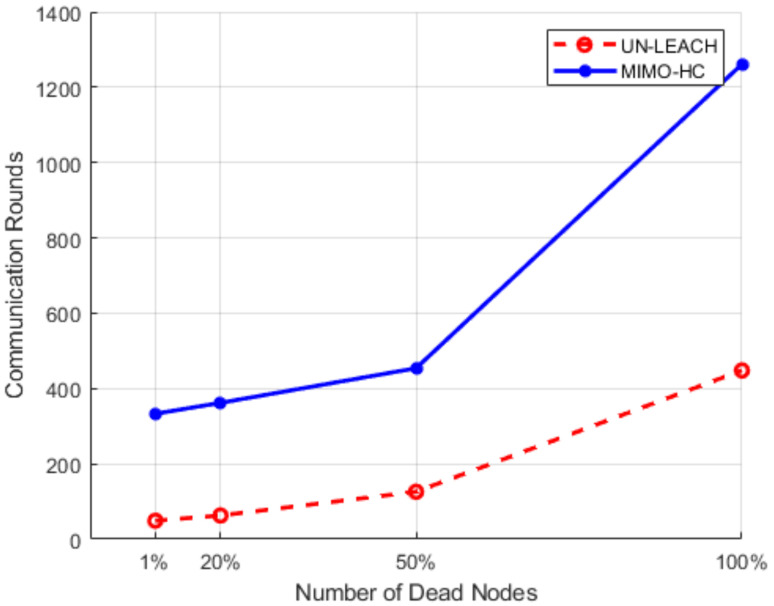
Performance results for 150 nodes in a 200 m × 200 m network.

**Figure 14:**
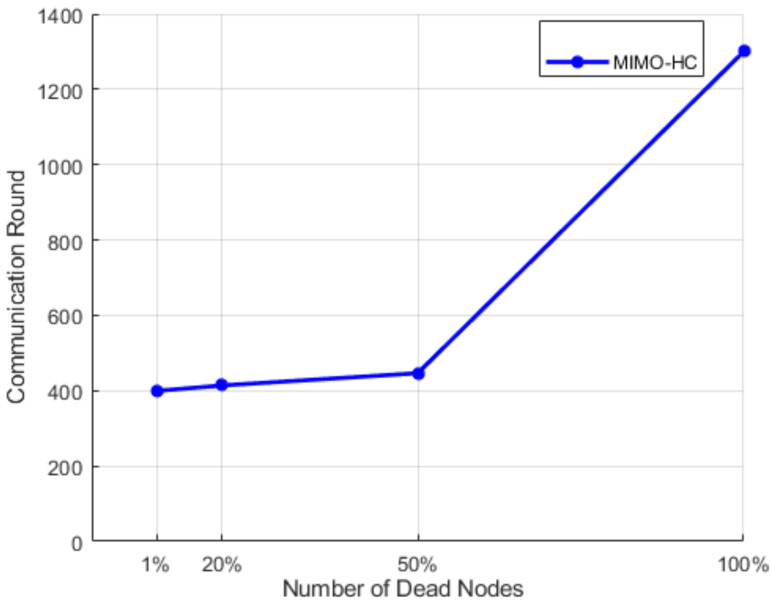
Performance results for 300 nodes in a 200 m × 200 m network.

Finally, Figure 13 depicts the number of communication rounds in an area of 200 m × 200 m when a 1, 20, 50, and 100% of sensors perishes. Sensors at this area start to fall at a faster pace after around 20% of the sensors have perishes. This is attributed to the large range among the sensors, and each sensor node wants to be a leader head. This induces a quick energy consumption. The number of rounds in MIMO-HC is approximately 3 times more than in UN-LEACH [11], when 1, 20, 50, and 100% of sensors perishes for a zone size of 200 m by 200 m. In addition, Figure 14 displays the result of an area of 200 m × 200 m with 300 node density (i.e., with an additional 150 nodes) when a 1, 20, 50, and 100% of sensors perish. It shows that the network in the proposed MIMO-HC is scalable and has a longer network lifetime than the network with only 150 nodes. This is, therefore, the additional nodes deployed can be regarded as increasing the total energy of the network.

## 6. Conclusions

This article proposes a centralized approach for organizing Internet of Things communication entities into an unequal hierarchy of hybrid clusters with the goal of preserving the network from rising hotspot issues and extending the lifetime of the network under the 5G environment. In particular, the suggested protocol has unique characteristics such as chain topology formation, clusters of different communication topology, balancing energy consumption among cluster heads, preserving energy consumption of nodes within the cluster, selecting and implementing suitable communication interface of the IoT application system. These features were achieved through the following implementations. First, a vector projection approach that takes into account the central station position for generating shorter concentric chains was used. In addition, a probability sub-optimal and multi-hop routing mechanism were implemented to lessen the load on the cluster heads and to extend the lifespan of the network. Finally, a ranking method was used to select proper communication interface. Numerical results showed that unequal clustering mechanism optimized the existence of network and balanced energy depletion through cluster heads benchmarked against the state-of-the-art techniques.

In addition to the numerical simulations, as a future research direction, the proposed energy-efficient routing protocol can be augmented with a real-world off scenario-based framework. For instance, a co-simulation of the wireless sensor node deployment can be conducted by incorporating their geographic coordinates assuming the sensor nodes are GPS-enabled.

## Figures and Tables

**Table 1:** Comparison of our proposed scheme with existing state-of-the-art literature interfaces.

Lit.	Approach	Main Idea	Max Lifetime	Load Balancing	Cluster Formation Overhead	Fault Tolerance
[6]	LEACH	Randomly chooses several sensor nodes as Cluster Head and circulates this position between the nodes within a cluster	✓	Moderate	⇑	X
[7]	PEGASIS	Greedy algorithm is used to construct the chain. Cluster Head at each round is selected randomly	✓	Moderate	↓	X
[8]	EEUC	Cluster heads are elected based on a Competition Radius, were clusters radius is designed to be commensurate to their range to central station	✓	good	⇑	X
[11]	Unequal LEACH	Distance matrix created to modify transmission power. Distance and residual energy to central station considered for cluster head election	✓	good	⇑	X
Our paper	MIMO-HC	Cluster heads are elected based on residual energy and distance to the central station. Sub-optimal rout generated between cluster heads proportional to their range to the central station	✓	very good	⇓	✓

✓ supports or shows capability, ⇑ high transmission overhead, ⇓ low transmission overhead, ↓ average transmission overhead, X does not support or no capability.

**Table 2:** Simulation parameters of MIMO-HC.

Parameter	Value
Network coverage	(0, 0)–(200, 200) m
Central station location	(100, 250) m
N	150
$E_{elec}$	50 nJ/bit
$\epsilon_{fs}$	10 pJ/bit/m${}^{2}$
$\epsilon_{mp}$	0.0013 pJ/bit/m${}^{4}$
$E_{DA}$	5 nJ/bit/signal
Initialenergy	1 J
Interfaces	WiFi, Bluetooth, Zigbee, LTE
$d_{o}$	40 m
BroadcastPacketSize	25 bytes

## Data Availability

Data sharing not applicable.
